# Grain Disarticulation in Wild Wheat and Barley

**DOI:** 10.1093/pcp/pcac091

**Published:** 2022-06-28

**Authors:** Mohammad Pourkheirandish, Takao Komatsuda

**Affiliations:** School of Agriculture and Food, Faculty of Veterinary and Agricultural Sciences, University of Melbourne, Royal Parade, Parkville, Melbourne, VIC, 3010, Australia; Crop Research Institute, Shandong Academy of Agricultural Sciences, Jinan, Shandong 250100, China

**Keywords:** Brittle rachis, Domestication, Neo-functionalization

## Abstract

Our industrial-scale crop monocultures, which are necessary to provide grain for large-scale food and feed production, are highly vulnerable to biotic and abiotic stresses. Crop wild relatives have adapted to harsh environmental conditions over millennia; thus, they are an important source of genetic variation and crop diversification. Despite several examples where significant yield increases have been achieved through the introgression of genomic regions from wild relatives, more detailed understanding of the differences between wild and cultivated species for favorable and unfavorable traits is still required to harness these valuable resources. Recently, as an alternative to the introgression of beneficial alleles from the wild into domesticated species, a radical suggestion is to domesticate wild relatives to generate new crops. A first and critical step for the domestication of cereal wild relatives would be to prevent grain disarticulation from the inflorescence at maturity. Discovering the molecular mechanisms and understanding the network of interactions behind grain retention/disarticulation would enable the implementation of approaches to select for this character in targeted species. *Brittle rachis 1* and *Brittle rachis 2* are major genes responsible for grain disarticulation in the wild progenitors of wheat and barley that were the target of mutations during domestication. These two genes are only found in the Triticeae tribe and are hypothesized to have evolved by a duplication followed by neo-functionalization. Current knowledge gaps include the molecular mechanisms controlling grain retention in cereals and the genomic consequences of strong selection for this essential character.

## Introduction

**Fig. 1:**
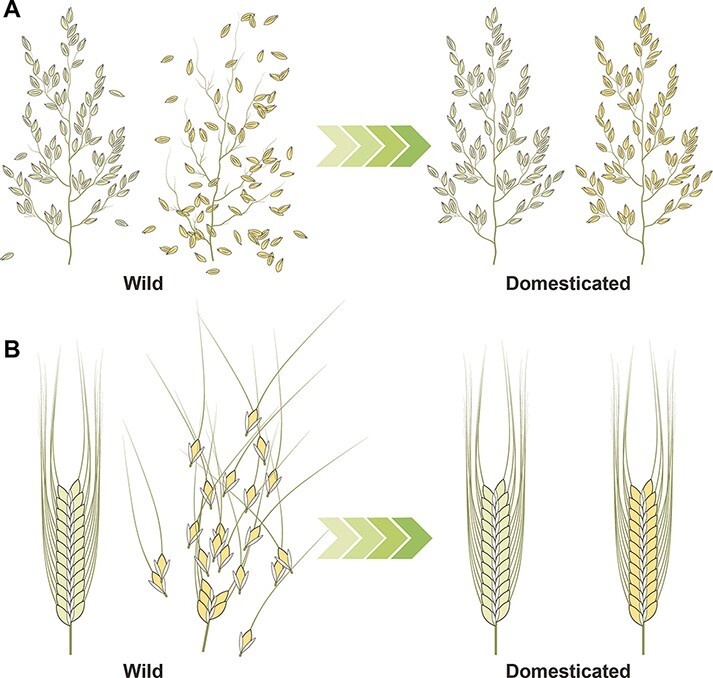
Stable harvest is a key factor in crop domestication. (A) In wild rice with panicle inflorescence, seeds break above the glume and shatter at maturity where all rachis remain on the panicle. Non-shattering trait was selected during rice domestication. (B) In wild barley and wheat with spike inflorescence, grain is separated from spike with a rachis segment attached and falls to the ground. The trait is called the brittle rachis. Domesticated barley and wheat retain the grain on the spike, and the whole spike can be easily harvested, also known as non-brittle rachis.

Plant domestication was a major pillar in the transition from hunter-gatherer to agriculture-based societies. Domesticated crop species underwent a series of morphological changes that today enable farmers to sustain a population of over 7.2 billion people. As a part of international efforts to bring about a second Green Revolution with a focus on low input and high nutritional output, the domestication of new crops is being considered (Osterberg et al. 2017). Understanding the fundamental morphological changes that historically enabled crops to become domesticated will help to improve our future crops to feed an expanding world population in an era of climate change (Pourkheirandish et al. 2020). Perhaps the most critical event in the process of cereal domestication was the loss of the natural mode of grain dispersal (Salamini et al. 2002). All wild plant species have grain dispersal mechanisms; however, the separation position of grain from the inflorescence varies among different genera (Doust et al. 2014). Grains from wild cereals such as wheat and barley progressively break off along the inflorescence structure, the spike, and scatter on the ground as the plant senesces and dries. During this process, the central axis of the inflorescence, the rachis, becomes brittle and snaps; the wild-type phenotype is therefore referred to as ‘brittle rachis’ (**Fig. 1**). One of the first steps in domesticating a grain-producing plant was selection against grain shattering. Early farmers naturally selected plants that had lost this trait and retained the grain on spikes, making grains easy to harvest.

Wheat and barley with a stiff (non-brittle) spike have been selected independently several times by early farmers who settled east of the Mediterranean Sea. In barley, independent mutations in two genes responsible for grain disarticulation, called Non-brittle rachis 1 and Non-brittle rachis 2 (btr1, btr2), have been selected during the process of domestication (Takahashi and Yamamoto 1949, Pourkheirandish and Komatsuda 2007). Both functional genes are required for disarticulation. Mutation in either gene can cause cell wall thickening in the grain separation zone, making the rachis non-brittle so as to retain grain (Pourkheirandish et al. 2015). It has been demonstrated that orthologous genes control grain dispersal in wheat (Avni et al. 2017, Pourkheirandish et al. 2018, Nave et al. 2019). These two genes’ orthologs are absent in Arabidopsis, rice and even maize and only recently evolved in a tribe of cereals including wheat and barley, Triticeae. However, the molecular mechanism conditioning the process of grain separation from the spike for brittle rachis genotypes and the role of BTR1 and BTR2 proteins is not well understood.

Paralogs of *Btr1* and *Btr2* were discovered in wheat and barley named *Btr1*-*like* and *Btr2-like* genes. The *Btr* genes are limited to certain cereal species closely related to wheat and barley, whereas *Btr-like* genes are present more widely in grass family (Zeng et al. 2020). The biological and molecular functions of *Btr-like* genes were investigated to infer *Btr* function and divergence (Cross et al. 2022). This study suggested that *Btr* and *Btr-like* genes result from a segmental duplication. *Btr-like* genes conserved the original function of cell wall development in immature anthers, whereas *Btr* genes gained a different role related to the cell wall development at the immature rachis nodes, resulting in a novel mechanism of grain disarticulation.

In this review, we aim to leverage the original discoveries of genes controlling grain dispersal (brittle rachis) in wheat and barley to investigate the gene network and suggest further studies to find underlying molecular mechanisms controlling this phenotype crucial to evolution and domestication. Understanding the molecular genetics of grain retention will not only yield fascinating insights into a plant trait that supports human civilization and survival but will also underpin efforts to improve crops through introgression of useful genes from wild species and in the domestication of entirely new cereal crops.

## Brittle Rachis Evolved in the Triticeae Tribe

An inflorescence is a cluster of flowers arranged on a stem (rachis). Grass inflorescences are developmentally diverse. Inflorescence branching depends on the developmental fate of the axillary apical meristem and produces various patterns in different species. The final architecture of the inflorescence is the overall sum of the total number of meristems and their arrangement. It is believed that the panicle (a highly branched inflorescence as in rice) is a primitive form from which the spike (an unbranched inflorescence as in wheat) evolved (Judd et al. 1999) (**Fig. 1**). In wild wheat and barley species, upon maturity, the individual units of the spike break at the rachis between the spikelets and disperse the grain with a segment of the rachis, called the brittle rachis. While a brittle rachis is perfectly suited to optimize grain dispersal in nature, picking up individual grains from the ground makes harvesting difficult and is not desirable in agriculture. In contrast, mature grain from domesticated cereals is harvested easily and efficiently because the rachis is non-brittle, and the grain remains attached (**Fig. 1**).

**Fig. 2:**
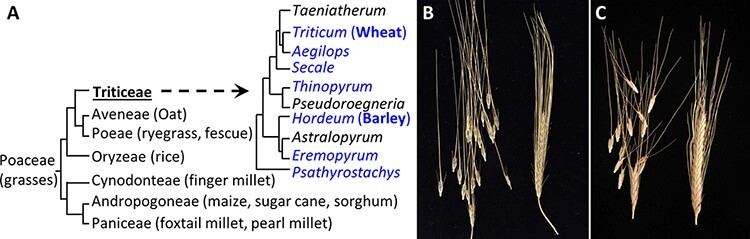
Grass spike evolution. (A) Evolution of the grasses with detail on the Triticeae branch. Members with brittle rachis are in blue (adapted from Judd et al. 1999 and Devos 2005). (B) Barley spike showing the brittle rachis of wild barley line OUH602 (Left) and its non-brittle rachis mutant M96-1 (Right). (C) Wheat spike showing the brittle rachis of wild einkorn (Tb) accession KT1-1 (Left) and non-brittle rachis of domesticated einkorn (Tm) accession KT3-5 (Right) (adopted from Pourkheirandish et al. 2018).

The Triticeae, the wheat tribe of grasses, includes economically important crops such as barley, rye, wheat and their wild relatives (**Fig. 2**) (Devos 2005, Bernhardt et al. 2017). This tribe harbors more than 350 species (30 genera). Bread wheat is an allopolyploid species that originated by interspecific hybridization of three different diploid species and subsequently contains three ancestral genomes termed; A, B and D. Two of the progenitor species (A and B genomes) exhibit a brittle rachis at maturity, like wild barley. Wild *Hordeum* (barley), *Triticum* (wheat), *Secale* (rye), *Taeniatherum* and *Thinopyrum* have spike inflorescences with brittle rachis disarticulation (**Fig. 2**). There are also several that have spike inflorescences with brittle ‘rachilla’ disarticulation genera, including *Australopyrum, Anthosachne, Elytrigia, Elymus* and *Leymus*. The mechanism of grain disarticulation driven by *Btr1* and *Btr2* is only found in the Triticeae tribe (Sakuma et al. 2011). The exclusive inflorescence structure (spike), unique separation mechanism (brittle rachis) and newly evolved genes (*Btr1*/*Btr2*) are common properties only found among the Triticeae (Zeng et al. 2020). These factors collectively support the hypothesis that the brittle rachis evolved relatively recently within the Triticeae tribe.

The non-brittle rachis results from a mutation in either *Btr1* or *Btr2* in barley. These non-brittle types have arisen at least two times in barley of either a 1-nucleotide deletion at *Btr1* coding sequence resulting in frameshift originating from wild barley collected from southern Levant (Israel, Jordan) or a 11-nucleotide deletion at *Btr2* coding sequence resulting in frameshift originating from wild barley collected from Northern Levant (Syria, south Turkey) (Pourkheirandish et al. 2015). One amino acid substitution in the BTR1 protein found in cultivars originating from wild barley from the Gaziantep region of south-east Turkey was suggested as an independent origin. However, unequivocal genetic evidence to demonstrate that it is responsible for the non-brittle rachis is yet to be shown (Civan and Brown 2017). Non-brittle wheat was also selected from the mutation at the *Btr1* coding sequence due to an amino acid substitution in diploid wheat (einkorn) evident from south-east Turkey (Pourkheirandish et al. 2018), a 2-nucleotide deletion at *Btr1-A* coding sequence resulting in a frameshift and a 11-nucleotide deletion at the promoter region of *Btr1-*B in tetraploid wheat (Avni et al. 2017, Nave et al. 2019).

## How Do *Btr1* and *Btr2* Control Cell Wall Thickness in the Separation Layer?

**Fig. 3:**
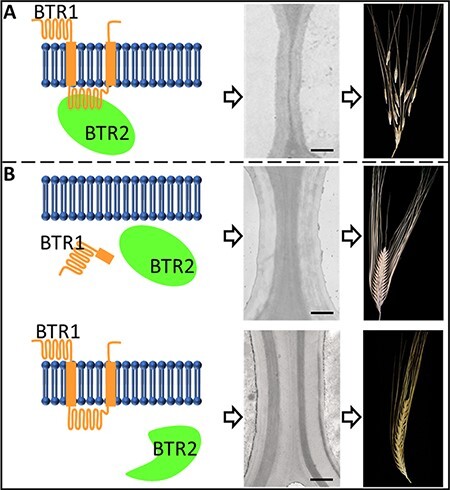
Model of the interaction between BTR1 and BTR2 proteins. It is proposed that BTR1 acts as a receptor and BTR2 as a ligand. (A) The BTR1–BTR2 interaction results in thin cell walls and grain dispersal at maturity. (B) Loss of function of either protein impairs the interaction and results in thick cell walls and an intact spike even at maturity.

Previous studies have shown that functional BTR1 and BTR2 proteins are required for disarticulation of spikelets from the spike in both wheat and barley (Pourkheirandish et al. 2015, 2018). However, we do not know ‘how’ they control this process. We know that they promote the formation of thinner cell walls in a so-called separation layer, but we do not understand why or how this is elaborated. However, cytological observations suggest a reprogramming of local primary and/or secondary cell wall formation. Why this occurs only in the cells of the separation layer is unknown. The in silico analysis predicted that BTR1 is a membrane protein containing two transmembrane helices while BTR2 was cytoplasmic (Pourkheirandish et al. 2015). Based on these subcellular locations, we can hypothesize that BTR1 and BTR2 potentially encode a receptor–ligand pair constituting key components of a signal transduction pathway that initiates the highly localized cellular response (**Fig. 3A**). While this model is likely, we have no ‘proof’ of their subcellular location or whether the receptor–ligand hypothesis is correct. Furthermore, the upstream signal(s) that promote localized, temporal *Btr1/2* expression, restricting the control of cell wall thickness strictly to the separation layer, and the consequences of the signal transduction response operating through *Btr1/2* are unknown.

Brittle rachis is a unique character that evolved in Triticeae; thus, it is necessary to use a Triticeae member such as wheat or barley to study the function of *Btr1* and *Btr2* genes (Kellogg 2021). These materials will allow direct comparisons between plants that show the brittle rachis phenotype and plants that do not, in the same genetic background (Pourkheirandish et al. 2015) that can illuminate the molecular function of brittle rachis. Wild barley genotype OUH602 (brittle type) can be compared to its non-brittle sodium azide-induced mutant M96-1, which has a premature stop codon in the *Btr1* gene causing grain retention to test the biological hypotheses related to the molecular function of BTR1 and BTR2. Results can be validated in multiple independent genotype pairs of domesticated cultivars: (i) a non-brittle Golden Promise control (*btr1Btr2*) can be compared with the brittle transgenic line of Golden Promise (*btr1Btr2 *+ p*Btr1:Btr1*), where brittleness was restored by the introduction of a functional *Btr1* allele, and (ii) a non-brittle recombinant inbred line RIL50 (*Btr1btr2*) can be compared to a brittle transgenic line of RIL50 (*Btr1btr2 *+ p*UBI1:Btr2*), where brittleness was restored by introduction of a functional *Btr2* allele. The induced mutant and transgenic lines produced in the previous study can be an immediate target for further analysis (Pourkheirandish et al. 2015).

## The Brittle Rachis Signaling Pathway

Both BTR1 and BTR2 must be functional for grain disarticulation to happen. Given these two proteins’ structural predictions, it is hypothesized that BTR1 and BTR2 physically interact, potentially forming a receptor::ligand pair. The protein–protein interaction hypothesis will demonstrate the molecular interaction between BTR1 and BTR2 and can be tested using complementary approaches to determine if the two proteins directly interact to trigger a common signaling pathway or work independently but are still involved in a shared signaling network (Lampugnani et al. 2018, Zheng et al. 2019). A split ubiquitin yeast two-hybrid assay would test for interactions between the membrane protein BTR1 and its putative ligand BTR2 (Li et al. 2016). Transgenic barley plants expressing BTR1 and BTR2 proteins with fluorescent tags can be used to validate the yeast two-hybrid results in a native environment. Switch from non-brittle to brittle phenotype in transgenic lines will confirm the biological function of the target proteins. These will allow both functionality and localization to be investigated further. An overlapping signal will be expected if and where the two proteins co-localize. The underlying assumption is that the fluorescent tag does not interfere with protein complex formation and that it can be tested via phenotypic complementation. In the case of fusion protein interference, peptide antibodies can be raised against BTR1 and BTR2 to reveal the subcellular location of the respective proteins.

Co-localization of BTR1 and BTR2 will support the hypothesized molecular function of each protein and confirm their interactions. Finding other proteins involved in the brittle rachis phenomena will shed light on the molecular function of the grain disarticulation in spike. Even though BTR1 and BTR2 are specific to Triticeae, the cell wall thickness adjustment at the separation zone is part of a molecular pathway that can be specific to Triticeae or, more generally, exist in other plant species (Gorshkova et al. 2018, Anderson and Kieber 2020). Potential protein partners involved in this pathway should enable us to map these unique proteins in a functional context within the cell and engineer the pathway to open new research opportunities.

The expression of *Btr1* and *Btr2* has been detected in the developing barley inflorescence (white anther stage) using quantitative PCR (qPCR), and by using RNA in situ hybridization, *Btr2* mRNA has been detected in the abscission zone primordia (Pourkheirandish et al. 2015). However, the *Btr1* in situ hybridization was not successful in barley. It is unknown whether the expression of both genes was restricted to the separation layer or whether the separation layer was a region of overlap between wider expression domains of both genes. In *Aegilops longissima,* one of the wild relatives of wheat, *Btr1* was expressed broadly along nodes and internodes, rather than nodes specifically as observed in *Btr2* (Zeng et al. 2021). The subcellular protein localization will reveal further hints associated with their molecular operation. Immunohistochemistry can be used to characterize the BTR1 and BTR2 protein localization in wild barley. Transgenic materials mentioned above can be used to validate protein localization.

Based on the receptor–ligand hypothesis, BTR1 and BTR2 will likely exert their effect by triggering a developmental response that leads to altered cell wall structure in the disarticulation zone. Comparing expression profiles between brittle and non-brittle should elaborate on possible downstream genes activated by the BTR1–BTR2 signaling pathway. RNA sequencing of immature spike samples based on the expression time of the *Btr2* gene between the wild barley OUH602 (brittle type) compared to its induced mutant M96-1 (non-brittle) will identify potential downstream targets of *Btr1/Btr2*. This will reveal genes triggered by the reconstituted BTR1/BTR2 protein complex. These downstream targets will help understand the BTR initiation and phenotype signaling pathway.

Every genetic study has reported the *Btr* locus located on the short arm of chromosome 3 as major genes controlling brittle rachis in barley and wheat. Minor quantitative trait loci (QTL) affecting the brittle rachis trait independent of the *Btr* locus were also detected in different genetic investigations (Komatsuda and Mano 2002, Kandemir et al. 2004, Komatsuda et al. 2004). Some of these independent loci may act as a molecular partner of *Btr* genes, and they may operate in the brittle rachis development pathway. The genomic location of historical QTL discovered in association with brittle rachis can be instrumental in studying the molecular mechanism of grain disarticulation.

## What are the Physical and Compositional Changes in the Walls of Cells that Promote Disarticulation?

It has been demonstrated that changes in cell wall thickness are strongly associated with grain dispersal in barley at maturity (Pourkheirandish et al. 2015) (**Fig. 3A, B**). Histochemical staining of rachis sections revealed that five to six cell layers expand above each rachis node in brittle lines (*Btr1Btr2*) but not in non-brittle types (*btr1Btr2* or *Btr1btr2*). Fluorescence staining suggested a significant reduction in lignin content in the separation layer (abscission zone) below the rachilla node in species such as wild rice, *Bromus* spp. and *Agropyron* spp. that disarticulate below the rachilla node (Pourkheirandish et al. 2015). A follow-up study demonstrated lignin reduction at the rachis node in *Aegilops tauschii* that disarticulate below the rachis node (Zeng et al. 2020a). No lignin reduction was detected in wild barley. It remains to be examined whether the thinning of the cell wall at the separation zone in wild barley is due to (i) the regulation of primary and/or secondary wall biosynthesis or (ii) changes to specific cell wall components that alter the overall composition of this region. These fundamental questions about whether a specific component is missing in the separation zone or a simple proportional reduction in cell wall thickness responsible for rachis breakage will provide clues about genes involved in the brittle rachis process.

Plant cells are surrounded by a wall of rigid extracellular fibrils embedded in an amorphous matrix. Cell walls typically occur in two forms: a flexible primary wall while the cell is growing and a secondary wall that increases the strength of the wall and reduces flexibility (Evert and Eichhorn 2006). The primary wall has a high matrix polysaccharide content with cellulose but lacks lignin, whereas secondary wall thickening includes depositions of crystalline celluloses, heteroxylans and lignins (Evert and Eichhorn 2006).

Cell wall formation and subsequent modifications in the developing barley spike can be monitored by sampling at different developmental stages starting from the awn primordium stage when the spike is about 5 mm in length to full maturity (Kirby and Appleyard 1981). To observe and quantify cell wall thickness in a spatiotemporal manner comparing brittle and non-brittle types, transmission electron microscopy should be employed to image the cell wall layers at different stages. This fundamental experiment will show when cell wall thickness changes and enable a comparison of the separation zone and non-separation zones between wild type and mutants and the type of cell wall—primary, secondary or both—modified. The study will test the basic question of whether the thin cell wall results from the suppression of cell wall biosynthesis or is the consequence of degradation of developed walls similar to that observed in wild rice (Thurber et al. 2011).

The specific cell wall component changes between brittle (wild type) and non-brittle (induced mutant) types should be further examined. Fluorescence-based immunohistochemical studies using specific antibodies against cell wall polysaccharides, including arabinoxylan (LM11), xyloglucan (LM15), (1,3;1,4)-β-glucan (BG1), de-methylesterified pectin (LM19) and methylesterified pectin (LM20), as well as the cellulose-binding module (CBM3a) can be used at the rachis junction to investigate the wall composition in situ. This will provide a spatiotemporal map of where and when wall changes occur in the rachis junction.

## The Origin of Brittle Rachis

The *Btr1* and *Btr2* genes controlling grain disarticulation in wild wheat and barley are specific to Triticeae. How the *Btr* genes evolved and gained the brittle rachis function has been debated (Zeng et al. 2020, Cross et al. 2022). Basic Local Alignment Search Tool searches of the *Btr1* and *Btr2* sequences against the barley genome detected homology with two further hypothetical proteins termed BTR1-LIKE and BTR2-LIKE (Pourkheirandish et al. 2015). The genomic organization and orientation of *Btr1*/*Btr2* and *Btr1-like*/*Btr2-like* gene pairs in barley and wheat are exactly the same (Pourkheirandish et al. 2018), suggesting that the configuration must already have been present in their common ancestor before the divergence of the wheat and barley lineages dating back to more than 8 million years (Middleton et al. 2014).

**Fig. 4:**
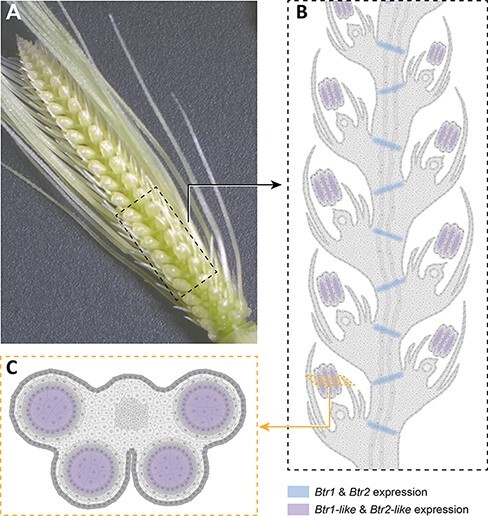
Model of *Btr* and *Btr-like* gene expression: a hypothesis to explain their mechanism of action. It is proposed that *Btr* and *Btr-like* genes are expressed in the same stage of the immature spike. (A) The barley immature spike at the white anther stage. (B) Schematic longitudinal section of the spike section is shown in the black broken rectangle. *Btr* genes are expressed at rachis primordia shown with blue highlight. This is supported by *Btr2* in situ hybridization data although *Btr1* in situ hybridization failed (Pourkheirandish et al. 2015). *Btr-like* genes are expressed at anther primordia shown with purple highlight. This is supported by RNA-seq expression data, which showed that *Btr1-like* and *Btr2-like* genes are exclusively expressed at the immature anther (Cross et al. 2022). Direct evidence by in situ hybridization for *Btr1-like* and *Btr2-like* remains to be investigated. (C) Schematic horizontal section of an anther shown in the orange broken rectangle and *Btr-like* genes expression shown in purple.

With more than 55% amino acid identity between BTR1 and BTR1-LIKE also BTR2 and BTR2-LIKE, they are not simply redundant. A functional *Btr1-like* gene cannot recover non-brittle rachis caused by mutation at the *btr1* locus, and similarly, the mutated *btr2* locus cannot be compensated by a functional *Btr2-lik*e gene (Pourkheirandish et al. 2015). *Btr1-like*/*Btr2-like* pairs are exclusively expressed at the immature anther stage (Cross et al. 2022). Interestingly, the expression time coincides with the *Btr1*/*Btr2* expression, meaning that at the same immature spike stage, *Btr1-like*/*Btr2-like* is expressed at anther primordia, whereas *Btr1*/*Btr2* is expressed at separation zone primordia. It is hypothesized that *Btr1*/*Btr2* originated through a segmental duplication followed by divergence at the expression location. This hypothesis is demonstrated in **Fig. 4**. In situ hybridization of *Btr1-like* and *Btr2-like* are required to test this hypothesis. According to this assumption, *Btr1-like*/*Btr2-like* preserved the original biological function related to anther development and *Btr1*/*Btr2* gained a different biological function, which is grain disarticulation. The molecular function of BTR1-LIKE/BTR2-LIKE could be the same as BTR1/BTR2 proteins and the biological divergence could be just due to the shift of expression location. However, BTR1/BTR2 proteins may gain a different molecular function. BTR1/BTR2 may cooperate in dissolving a cell wall component essential for the rigidness of the wall at maturation of the rachis; BTR1-LIKE/BTR2-LIKE may cooperate similarly, but in the anther cell wall surrounding the microspore cells. In contrast, BTR1/BTR2 may interfere with the cell wall biosynthesis at the rachis junction, resulting in a thinner cell wall that can break easier at maturity, while BTR1-LIKE/BTR2-LIKE may act alike in cell wall wrapping microspore cells. Co-expression analysis suggested that *Btr-like* genes involved in the cell wall biosynthesis and control of cell wall development are synergic with BTR1/BTR2 operation. Here we hypothesize that *Btr* and *Btr-like* molecular networks may overlap, but this requires further investigation. The brittle rachis mechanism evolved through a duplication followed by neo-functionalization, which is described in the case of other genes/traits (Pourkheirandish et al. 2007, Sakuma et al. 2010, Lye and Purugganan 2019).

## Back to the Future: Re-domestication of Non-brittle Barley from Wild

Ancient farmers selected non-brittle wheat and barley due to the ease of harvest. Once these mutants were fixed, growers always selected against brittle rachis which has resulted in a strong bottleneck effect at these loci. Plant breeders often intercross domesticated crop species with their wild relatives to bring across one or a few small chromosomal segments from the wild species to the domesticated recipient to improve cultivars with desirable traits (He et al. 2019). However, a huge diversity and genetic potential of wild grasses are left behind (Pourkheirandish et al. 2020). The opposite procedure where the wild progenitor can be used as a recurrent parent with selection against domestication traits has recently been suggested (Langridge and Waugh 2019). In this way, we back to the original resources to re-domesticate barley or wheat, taking advantage of currently known domesticated genes. The knowledge generated by understanding the domestication process could be used to modify wild relatives of grain crops and forage species that are fit for specific environments. Many agronomically adapted forage species are commercially unavailable due to poor seed production characteristics. It will be possible to look for mutations within brittle rachis genes using reverse genetic approaches and to use undirected mutagenesis approaches in the wild species to find mutants that retain grain on the spike. Perennial crop development is an emerging field of research focused on improved environmental sustainability of crop production system. Intact rachis is an incredibly important trait for perennial crops and forages as their flowering time is indeterminant and the grain needs to be retained longer on the earlier maturing heads. There are two pathways to new crop development; hybridization and domestication. Examples of perennial wild grass species that can be hybridized with wheat are members of the *Thinopyrum* genus. *Thinopyrum* is well known for high biomass production and high protein content as well as cold and salt tolerance and disease resistance. Species within this genus have already been widely used to cross-hybridize with wheat to transfer chromosomal segments containing genes for these valuable characteristics into wheat (King et al. 1997, Liu et al. 2013, Kuzmanović et al. 2014). An example of a perennial wild grass that proved more difficult to hybridize with wheat is *Elymus*, which is well known for its drought tolerance that is critical for the Australian environment (Newell et al. 2015). In this case, domestication might be a better approach. An alternative approach would be genome editing, which was recently applied to re-domesticate wild tomato as a proof of concept (Li et al. 2018, Zsögön et al. 2018). However, it is important to mention that currently there are no transformation protocols for most wild species even though there are efforts to overcome those barriers (Wang et al. 2022).

## Negative Consequences Associated with Selection for Grain Retention

Non-shattering was critical for barley domestication; as mutations in either *Btr1* or *Btr2* can result in grain retention, there were at least two domestication paths in barley. Despite the ability of mutations in either loci to result in grain retention, genetic diversity around the *Btr1* and *Btr2* loci is limited to one or two alleles at each locus (Pourkheirandish et al. 2015, Civan and Brown 2017). Low diversity around the *Btr* region is exceptional compared to other independent loci in barley. For example, six-row spike locus *vrs1* has at least eight alleles, including two-row and six-row type results from several allele exchanges between wild and cultivated forms at this locus (Saisho et al. 2009). The bottleneck effect at *Btr* loci can lead to two issues for barley improvement. Firstly, the introduction of novel diversity by crosses with wild barley has been successful for many regions and traits but has been impractical for the regions surrounding *Btr* due to strong selection for the mutant alleles. This probably hindered the introduction of beneficial alleles at linked loci. Secondly, negative traits associated with loci linked to the *Btr* loci remained fixed in cultivated barley. Several studies demonstrated QTL from wild barley in the vicinity of the *Btr* loci with positive effect in agronomic characteristics, including grain number per ear (Saade et al. 2016), grain elements such as B, K and Zn (Herzig et al. 2019), resistance against net blotch (Vatter et al. 2017) and microbiota composition in the rhizosphere (Escudero-Martinez et al. 2022). Other studies have indicated that linkage drag associated with key domestication or adaptive traits can significantly impact the overall crop performance (Dreissig et al. 2020).

It remains to be answered if selection for grain retention carries detrimental alleles through to cultivated barley. We know that *btr1* and *btr2* would have been under intense selection during domestication. They reside in a low-recombining region of the barley genome, potentially limiting the local diversity available to breeders (Pourkheirandish et al. 2015). Previously, we were able to discover recombination between *Btr1* and *Btr2* in a large segregating population (four recombinants out of 14,000 F_2_) (Pourkheirandish et al. 2015). Recombination also detected between *Btr* genes among the natural population of *agriocrithon* barley that has a six-row and brittle phenotype (Pourkheirandish et al. 2018a). These indicate that recombination between and around *Btr1* and *Btr2* is possible but at a low frequency. Comparing these recombinants will assess the implication of fixation on genetic diversity and develop options to bring novel variations into cereal breeding programs.

## Data Availability

New data were not generated in this research.
